# The *CONSTANS-like 2* Gene Serves as a Pivotal Regulator of Flowering in *Hemerocallis*

**DOI:** 10.3390/plants14131996

**Published:** 2025-06-30

**Authors:** Chunjing Guan, Yike Gao, Ziyi Wang, Qixiang Zhang

**Affiliations:** Beijing Key Laboratory of Ornamental Plants Germplasm Innovation & Molecular Breeding, National Engineering Research Center for Floriculture, School of Landscape Architecture, Beijing Forestry University, No. 35 Qinghua East Road, Haidian District, Beijing 100083, China

**Keywords:** *Hemerocallis*, flowering, circadian, *HfCOL*2

## Abstract

*Hemerocallis* spp. exhibit distinct flower opening times, categorized into nocturnal and diurnal types. Previous studies have demonstrated that the circadian clock and *CONSTANS* (*CO*) genes play crucial roles in regulating flowering in *Hemerocallis*. However, the key genes that integrate flowering pathways remain largely unknown. To address this gap, we identified potential homologs of the *FLOWERING LOCUS T* (*FT*) gene in *Hemerocallis*. A yeast one-hybrid assay revealed that HfCOL2 and HfLHY directly bind to the *HfFT1* and *HfFT2* promoters, thereby activating *FT* transcription. The expression analysis reveals that *HfCOL2* expression rhythms not only display opposing patterns between nocturnal and diurnal opening types of *Hemerocallis* but also between leaf and flower tissues. The peak expression of *HfCOL2* in flowers aligns closely with the respective opening times of diurnally and nocturnally flowering *Hemerocallis*. The overexpression of *HfCOL2* in tobacco plants led to early flowering and prolonged flower longevity. In *Hemerocallis*, the *HfCOL2* gene plays a pivotal role not only in photoperiod-induced flowering but also in the circadian rhythm-mediated regulation of flower opening time. Due to the limited availability of plant materials exhibiting distinct flower opening rhythms, research in this area has been constrained. Identifying the key genes in the flowering pathway of *Hemerocallis* can facilitate a better understanding of the mechanisms by which plants respond to circadian rhythms.

## 1. Introduction

Despite the absence of a central nervous system, plants have evolved complex mechanisms to perceive temporal and environmental changes, thereby enabling them to anticipate predictable alterations linked to the Earth’s rotation. These mechanisms collectively constitute the circadian system, which regulates a plant’s internal timing of temporal progression and orchestrates physiological and developmental processes to synchronize with variations in the environment [1]. By anticipating environmental changes, the circadian system optimizes the synchronization of plant physiology, metabolism, and growth, minimizing the lag between environmental cues and physiological responses [2]. The flowering process is regulated by this circadian system, which integrates external environmental signals and transmits them to the output pathway through its core oscillation system [3,4,5].

*CONSTANS* (*CO*) acts as a critical mediator that bridges the circadian clock with flowering control mechanisms, playing vital roles in various physiological aspects of growth and development, particularly regarding seasonal flowering [6,7]. In *Arabidopsis thaliana*, CO activates *Flowering Locus T* (*FT*) promoters by binding to CORE and CCACA cis-elements under long-day (LD) conditions, thereby promoting the development of floral organs. In rice, the *CO* homolog, *Heading date 1* (*Hd1*), enhances *Heading date 3a* (*Hd3a*, the *FT* homolog) expression under short-day conditions while repressing it in non-short-day environments [8,9,10]. The regulatory mode of the *CO*-*FT* pathway has been demonstrated across a variety of plant species [11,12,13,14,15,16,17].

Despite extensive research elucidating the molecular mechanisms underlying photoperiod-induced flowering in various plant species, our understanding of the circadian rhythm regulation governing the daily opening rhythms of flowers remains comparatively limited. The rhythmic opening and closing of flowers can be observed across various plant species, with the endogenous opening rhythm typically being determined by exposure to constant dark or light conditions [18]. Plants retain their inherent rhythm under constant environmental conditions, indicating that the circadian clock system maintains internal rhythm stability over extended periods [19,20]. Purslane (*Portulaca umbraticola*) flowers exhibit a sustained blooming rhythm lasting up to 3 days under continuous darkness, with synchronous blooming occurring in each flower [21]. Even under conditions of constant darkness, *Pharbitis* demonstrates a circadian rhythm during its flowering process [22]. Notably, flower opening displays robust rhythmicity under continuous light or darkness, reinforcing the involvement of the circadian clock in regulating this phenomenon [23]. In wild tobacco (*Nicotiana attenuata*), the disruption of flower opening rhythms was observed upon silencing clock genes *LHY* (*Late elongated hypocotyl*) and *ZTL* (*ZEITLUPE*) [19]. In the model plant *Arabidopsis thaliana*, flower opening is redundantly induced by the circadian clock through unknown light-sensing pathways, while flower closure is entirely dependent on circadian clock control [24].

Transcriptome sequencing analyses conducted at different flowering stages revealed that *EARLY FLOWERING 4-LIKE*, a key circadian clock gene, positively regulates flower opening in candy lily (*Iris* × *Norrisii*) [25,26]. Furthermore, transcriptome sequencing performed on hybrid offspring of *Hemerocallis* with distinct flower opening times revealed that pivotal genes such as *GI* (*GIGANTEA*) and *CO* are involved in regulating the floral rhythm of *Hemerocallis* [27,28]. Daily oscillations in *CO* mRNA are controlled by the circadian clock. In *Arabidopsis*, an oscillation rhythm of *CO* mRNA levels with a period of 24 h under LD conditions was identified, and the expression peak occurred at 16 h after dawn [6]. Ectopic expression of *CONSTANS-like 1* (*COL1*) and *CONSTANS-like 2* (*COL2*) genes impacted flowering time in *Arabidopsis*. Nevertheless, the overexpression of *COL1* was noted to shorten the periods of circadian rhythms [29]. In rice, three genes similar to *Arabidopsis* CONSTANS-like proteins (S12569, S3574, and C60910) exhibited transcriptional regulation by circadian rhythms. The oscillation period of the S12569 gene transcripts was approximately opposite to that of *OsGI* (the rice homolog of *GIGANTEA*) and *Hd1* (the rice homolog of *CONSTANS*) [30]. Homologs of the *CO* gene have also been identified in aquatic plant species of Lemna (*Lemna gibba G3*), where *LgCOH1* expression was induced by light, exhibiting diurnal rhythmic expression peaking during the day, suggesting a possible role for *COL* family genes in regulating the circadian system [31].

The limited availability of suitable materials has constrained the development of research on the circadian rhythm of flower opening to some extent. Additionally, current studies have yet to identify key genes that regulate this circadian rhythm. *Hemerocallis* spp., which exhibit distinct flower opening times and are classified as nocturnal and diurnal types, serve as an ideal model system for investigating the circadian rhythm regulation of flower opening. In a study of photoperiodic flowering induction in *Hemerocallis*, it was found that HfCOL2 binds to the promoters of *HfFT1* and *HfFT2*. Moreover, tobacco lines overexpressing *HfCOL2* exhibited an early flowering phenotype. Notably, the circadian rhythm expression pattern of *HfCOL2* showed opposite trends between flower and leaf tissues and among *Hemerocallis* species with different flower opening patterns. This study not only confirms that *HfCOL2* plays a critical role in photoperiod-induced flowering in *Hemerocallis* but also highlights its central function in regulating the circadian rhythm of flower opening. This discovery enhances our understanding of the mechanisms regulating plant flowering.

## 2. Result

### 2.1. *HfCOL2* and *HfLHY* Bind to the Promoter of HfFTs

**Figure 1:**
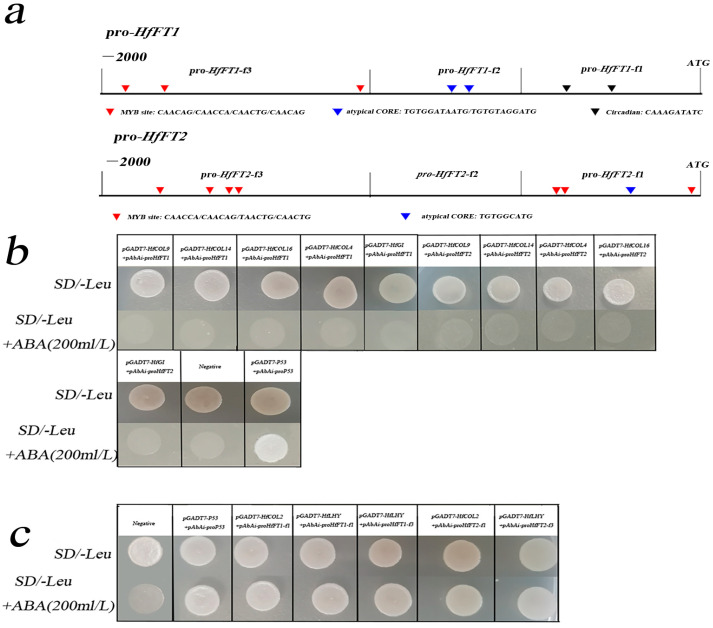
The interactions between HfGI, HfLHY, and CO-like proteins with the promoters of *HfFT1* and *HfFT2* were examined using the yeast one-hybrid (Y1H) assay. (**a**): Schematic representation of specific motifs in the *HfFT1* and *HfFT2* promoters. Different colored triangles indicate distinct putative binding sites. (**b**): Interaction analysis between the promoters of *HfFT1* and *HfFT2* and HfGI, HfLHY, HfCOL4, HfCOL9, HfCOL14, and HfCOL16. (**c**): Interaction analysis between the promoters of *HfFT1* and *HfFT2* and HfLHY and HfCOL2.

Previous studies have shown that the flowering of *Hemerocallis* is regulated by the central circadian clock genes *LHY* and *GI.* The signaling pathway is mediated through the *CO-like* gene family and subsequently transmitted to the *FT* gene, thereby achieving precise control over the flowering process in *Hemerocallis* [27]. To investigate the interactions between GI, LHY, and the CO-like family with the promoters of *FT*, we successfully amplified the promoters of the *HfFT1* and *HfFT2* genes using leaves from *Hemerocallis lilioasphodelus* L. Promoter sequence analysis revealed that the promoter regions of *HfFT1* and *HfFT2* contain unconventional Myb-family binding sites and circadian rhythm-related cis-elements, as well as CO binding sites (Figure 1a). These findings suggest potential interactions between the circadian clock, CO, and MYB family proteins within these promoter regions.

To determine whether HfGI, HfLHY, HfCOL2, HfCOL4, HfCOL9, HfCOL14, and HfCOL16 directly bind to these cis-elements in the promoter region of *HfFT1* and *HfFT2*, we conducted yeast one-hybrid (Y1H) assays. HfGI, HfCOL4, HfCOL9, HfCOL14, and HfCOL16 did not bind to the *HfFT1* and *HfFT2* promoters (Figure 1b). In contrast, HfLHY and HfCOL2 were able to bind to the *HfFT1* and *HfFT2* promoters, leading to the activation of the AbAir reporter in vitro (Figure 1c).

### 2.2. The Rhythmicity of Gene Expression over a 24 h Period

**Figure 2:**
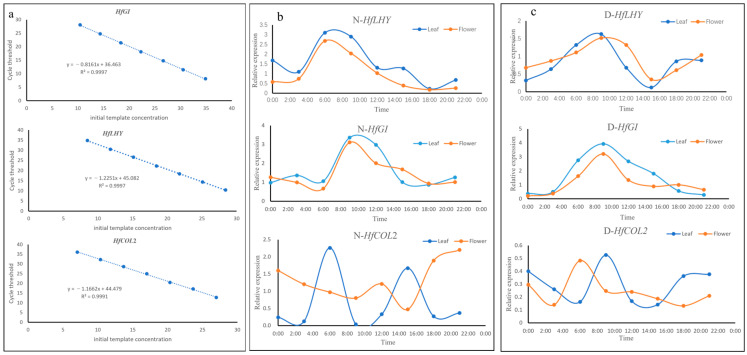
The 24 h expression rhythm of the *HfGI*, *HfLHY*, and *HfCOL2* genes under natural conditions in the flowers and leaves of nocturnal and diurnal flowering *Hemerocallis* species. D: Diurnal flowering; N: Nocturnal flowering. (**a**): Standard curve depicting the expression patterns of *HfGI*, *HfLHY*, and *HfCOL2* genes. (**b**): The 24 h expression rhythm of the *HfGI*, *HfLHY*, and *HfCOL2* genes in nocturnal flowering *H. lilioasphodelus*. (**c**): The 24 h expression rhythm of the *HfGI*, *HfLHY*, and *HfCOL2* genes in diurnal flowering *H. fulva*.

The expression levels of *LHY*, *GI*, and *CO* mRNA display a 24 h rhythmic pattern. Consequently, we examined the 24 h rhythmic expression patterns of *HfGI*, *HfLHY*, and *HfCOL2* in both leaves and flowers of *H. lilioasphodelus* and *Hemerocallis fulva* L. The cycle threshold (CT) values from the RT-qPCR reactions using specific primers for these genes were used as the ordinate to construct the standard curve. A ten-fold dilution series exhibited a robust linear correlation between the obtained CT values and template concentrations. The coefficient of determination (R²) fell within an appropriate range (Figure 2a).

*Hemerocallis lilioasphodelus* is a nocturnal-flowering species of *Hemerocallis*, with its flowers initiating bloom at 18:30 and reaching full bloom by 20:00. In contrast, *H. fulva* is a diurnal-flowering variety, with flowers beginning to open at 04:00 and achieving full bloom by 07:00. The expression of the *HfLHY* gene exhibited a distinct circadian rhythm. In the leaves of nocturnally flowering *Hemerocallis*, the expression peaked at 06:00, followed by a valley around noon (12:00), with a secondary smaller peak occurring at approximately 15:00. In contrast, in the leaves of diurnally flowering *Hemerocallis*, the expression peaked at 09:00 and then gradually declined, before rising again after 18:00. The *HfLHY* gene expression in nocturnally flowering *Hemerocallis* flowers reached its peak in the early morning (around 06:00) and subsequently decreased (Figure 2b). In diurnally flowering *Hemerocallis*, the *HfLHY* gene expression peaked later in the morning (around 09:00) (Figure 2c). These findings indicate that the *HfLHY* gene expression in both flower tissues of *Hemerocallis* species exhibits a characteristic morning peak. Moreover, the expression patterns in both leaves and flowers were consistent, with peaks occurring in the morning followed by a gradual decline.

In nocturnal-flowering *Hemerocallis*, *HfGI* gene expression in both leaves and flowers peaks around 09:00 and subsequently declines gradually (Figure 2b). In diurnal-flowering *Hemerocallis*, the *HfGI* gene expression patterns in leaves and flowers are similar, with a peak also occurring around 09:00, followed by a gradual decrease and remaining at low levels during the afternoon and evening (Figure 2c).

In the leaves of nocturnally-flowering *Hemerocallis*, the expression level of the *HfCOL2* gene peaked at 06:00, then gradually decreased, with a minor peak observed at 15:00; however, the overall expression remained lower than that in the morning (Figure 2b). In the flowers of nocturnally-flowering *Hemerocallis*, in contrast to the leaves, *HfCOL2* gene expression showed a minor peak at 12:00 and reached its highest level at 20:00 (Figure 2b). In diurnally-flowering *Hemerocallis* leaves, *HfCOL2* gene expression peaked at 09:00, then gradually declined, with a minor peak at 18:00 in the afternoon, but the overall expression level was relatively low (Figure 2c). In the flowers of diurnally-flowering *Hemerocallis*, unlike in the leaves, *HfCOL2* gene expression peaked at 06:00 (Figure 2c). The expression rhythms of the *HfCOL2* gene transcript exhibited opposite patterns not only between nocturnal and diurnal flowering plants but also between leaf and flower samples. The peak expression of *HfCOL2* in flowers aligns closely with the respective opening times of diurnally and nocturnally flowering *Hemerocallis*.

### 2.3. Overexpression of HfCOL2 Accelerates the Flowering of Nicotiana tabacum

**Figure 3:**
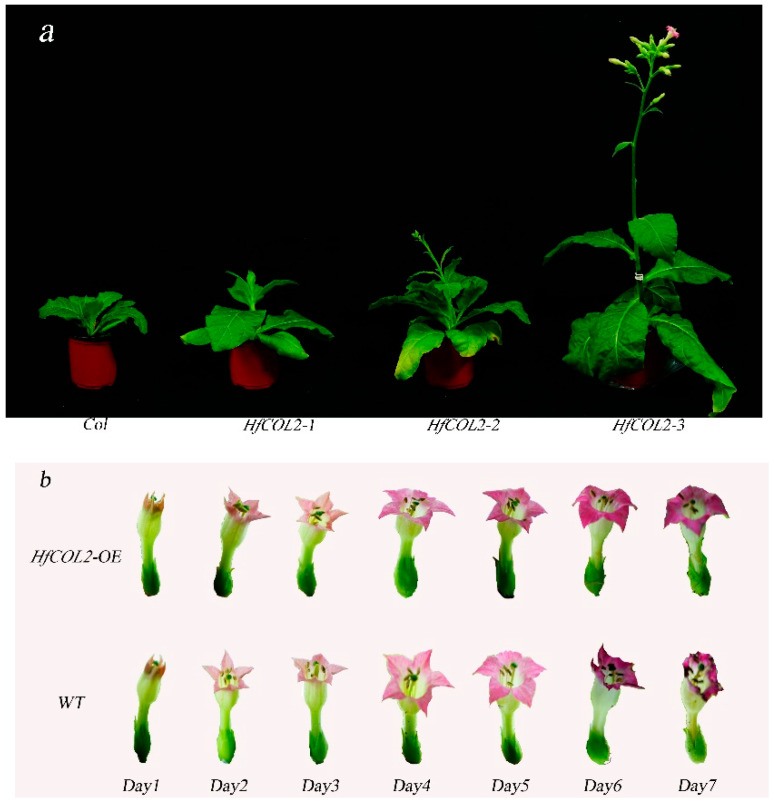
Overexpression of *HfCOL2* accelerates flowering in *Nicotiana tabacum* cv. K326. (**a**): Flowering time phenotypes of three *HfCOL2*-overexpressing transgenic lines compared to wild-type (Col) plants. (**b**): Comparison of the time of flower opening and senescence between wild-type (Col) and *HfCOL2*-overexpressing plants. *HfCOL2*-OE: Overexpression of *HfCOL2* transgenic lines; WT: wild-type *N. tabacum* cv. K326 plants.

The *HfCOL2* gene was successfully isolated from the leaves of *H. lilioasphodelus.* The full-length *HfCOL2* gene spans 786 base pairs and encodes a protein comprising 261 amino acids. Transgenic tobacco lines overexpressing the *HfCOL2* gene were generated. *HfCOL2*-overexpressing transgenic tobacco plants exhibited significantly earlier flowering times. A total of 48 days after sowing T1 seeds, the transgenic plants had either differentiated into flower buds or blossomed, whereas the wild-type tobacco plants had not yet developed any flower buds (Figure 3a). The mean flowering time of the transgenic plants was 49.90 ± 2.21 days, compared to 67.10 ± 2.16 days for the wild-type plants. Observations of the flower opening process in *HfCOL2*-overexpressing tobacco plants revealed a significant delay in flower senescence compared to wild-type tobacco. Specifically, *HfCOL2*-overexpressing transgenic lines exhibited a senescence time of 7 days post-anthesis, whereas wild-type tobacco flowers senesced at 6 days post-anthesis (Figure 3b). The *CO*/*COL* gene family is known to play a crucial role in regulating photoperiod-dependent flowering time in plants. These findings indicate that *HfCOL2* not only influences flowering time but also positively regulates the timing of flower senescence.

## 3. Discussion

Plants have evolved a sophisticated array of molecular pathways, including photoperiodic regulation, vernalization, and hormonal signaling cascades, to precisely coordinate the transition to floral initiation. These pathways converge on the flowering integrator gene *FT*, which regulates floral bud initiation [32,33,34]. Photoperiod regulation of flowering in *Hemerocallis* may involve two distinct pathways mediated by the *HfLHY*-*HfFT*s and *HfCOL2*-*HfFT*s modules. Specifically, HfLHY and HfCOL2 directly bind to the promoters of *HfFT*1 and *HfFT*2, thereby activating their transcription. In rice, two florigen genes, *Hd3a* and *RICE FLOWERING LOCUS T 1* (*RFT1*), have been identified. OsLHY can directly bind to the promoter of *RFT1*, while Hd1 can directly bind to the promoter of *Hd3a* [35,36,37]. There is homology in the flowering control mechanisms among monocotyledonous plants.

*HfCOL2* functions as a pivotal gene for photoperiodic flowering. HfCOL2 directly interacts with the promoter region of *HfFTs.* Overexpression of the *HfCOL2* gene in tobacco accelerates flowering while delaying senescence. Two *CONSTANS-like 2* homolog genes of mango (*Mangifera indica* L.), *MiCOL2A* and *MiCOL2B*, displayed distinct circadian rhythms and were highly expressed in leaves during the flowering induction period. The overexpression of *MiCOL2A* and *MiCOL2B* in transgenic *Arabidopsis* (Col-0) resulted in significant suppression of flowering [38]. In mungbean (*Vigna radiata*), *VrCOL2* exhibited daily oscillations in expression under short-day conditions. The overexpression of *VrCOL2* in *Arabidopsis* accelerated flowering under short-day conditions by modulating the expression of the flowering time genes *AtFT* and *AtTSF* [39]. In *Pharbitis nil*, the expression of *PnCOL1* exhibited both circadian rhythmicity and daily oscillation. The overexpression of *PnCOL1* driven by a *35S* promoter failed to rescue the late-flowering phenotype in *Arabidopsis CO* mutants, suggesting that *PnCOL1* may play a role in circadian regulation in *Pharbitis nil* [40]. In *Arabidopsis*, photoperiodic flowering is controlled by the regulatory hub gene *CONSTANS* (*CO*), which also plays a role in promoting flower senescence and abscission through the enhancement of jasmonic acid (JA) signaling and response [41].

The *HfCOL*2 gene plays a pivotal role in modulating the divergent flowering patterns of nocturnal-flowering and diurnal-flowering species within the genus *Hemerocallis*. In recent decades, extensive studies on *CO* and *COL* genes across various plant species have significantly enhanced our understanding of the molecular mechanisms governing flowering time regulation, stress responses, and root development [7,42]. The *CONSTANS* gene serves as a central regulator in the photoperiodic induction of flowering, exhibiting pronounced circadian rhythmicity across various plant species [7]. However, due to the scarcity of plant materials that display a distinct diurnal flowering rhythm, research on the regulation of the diurnal flowering rhythm by the *CO* gene remains in its infancy. Together, our data suggest that *CO* serves as a key component of the circadian rhythm that regulates flowering time. Investigating the mechanisms generating daily rhythms in *CO* mRNA levels and the potential role of light in post-transcriptional regulation of *CO* will provide deeper insights into how plants respond to daily rhythms.

## 4. Conclusions

In summary, the study demonstrates that the *HfCOL2* gene plays a pivotal role in regulating photoperiod-induced flowering in *Hemerocallis*. HfCOL2 directly interacts with the CORE cis-elements in the promoter regions of *HfFT1* and *HfFT2*, thereby modulating the flowering process and ensuring plants flower at the appropriate developmental stage. Furthermore, the *HfCOL2* gene plays a key role in the circadian control of flower opening in *Hemerocallis*. By elucidating the mechanisms governing daily fluctuations in *CO* mRNA levels, the study not only enhances the current understanding of plant responses to circadian rhythms but also provides a new way of thinking for investigating the complex interplay between genetic and environmental factors in shaping plant phenotypes.

## 5. Material and Method

### 5.1. Plant Material

The diurnal flower-opening species *H. fulva* and the nocturnal flower-opening species *H. lilioasphodelus* were cultivated in open-air conditions in the Changping District of Beijing, China (40°09′ N, 116°27′ E). Samples consisting of leaves and unopened flower buds of similar size from both species were collected at three-hour intervals throughout a single day, specifically at 00:00, 03:00, 06:00, 09:00, 12:00, 15:00, 18:00, and 21:00 h. Subsequently, the leaf and flower tissues were rapidly immersed in liquid nitrogen and stored at −80 °C until further analysis. Each sample was replicated three times to ensure circadian accuracy. The *N. tabacum* cv. K326 was used for the transgenic experiment. Plants were grown in an artificial climate chamber maintained at 25 °C (day/night), with a 16 h light/8 h dark cycle and 65% relative humidity.

### 5.2. RNA Isolation and Double-Strand cDNA Preparation

RNA isolation and double-strand cDNA preparation: The Quick RNA Isolation Kit (Beijing, China, Huayueyang) was utilized for RNA extraction from various samples, following the specific operational guidelines provided in the reference manual. The purity and concentration of the obtained total RNA were assessed through agarose gel electrophoresis and a Nanodrop2000 ultraviolet spectrophotometer analysis to ensure utilization of only high-quality RNA meeting specified criteria for subsequent reverse transcription experiments. Total RNA was reverse-transcribed into double-strand cDNA using the PrimeScript^TM^ RT kit with gDNA Eraser (Takara, Beijing, China), strictly adhering to the detailed instructions provided.

### 5.3. Yeast One-Hybrid Assay

For the cloning of *HfGI*, *HfLHY*, *HfCOL2*, *HfCOL4*, *HfCOL9*, *HfCOL14*, and *HfCOL16* using a cDNA template of *H. lilioasphodelus* leaves, RT-PCR amplification was performed using the primers listed in Table 1. The PCR conditions were as follows: initial denaturation at 94 °C for 1 min, followed by 30 cycles of annealing at 54 °C for 1 min, and extension at 72 °C for 1 min. The amplified fragments were subsequently analyzed by DNA sequencing (Qingke, Beijing, China).

In the cDNA template of *H. lilioasphodelus* leaves, PCR amplification was performed using primers with EcoRI and SacI restriction enzyme sites: *HfFT1*-EcoRI: CGGAATTCTTAAAAGCCAGTGTTAGGCCTGAAGA, *HfFT1*-SacI: GCGAGCTCTTCACGGTGATGGAGGGCTTGACTTC, *HfFT2*-EcoRI: CGGAATTCTTTGAACTCCCGACCTCTTGCTCACCTGC, and *HfFT2*-SacI: GCGAGCTCGCTTCACATCACACCCATTAATCACATTCG. Simultaneously, the pAbAi vector and *HfFT1* and *HfFT2* promoters were digested with the EcoRI and SacI restriction enzymes. The promoters were ligated to the pAbAi plasmid using T4 DNA ligase. Correct sequencing was verified by PCR, and the recombinant plasmid was extracted after successful verification. The recombinant plasmid was linearized using the BstBI enzyme and transformed into competent Y187 yeast cells. Transformants were plated on SD/-Ura medium and screened for resistance to different concentrations of AbA (Aureobasidin A) at 100 ng/mL, 150 ng/mL, 200 ng/mL, 500 ng/mL, and 700 ng/mL to determine the optimal inhibitory concentration.

Based on the aforementioned vector construction methods, the *HfGI*, *HfLHY*, and *CO-like* genes were cloned into the pGADT7 vector. The resulting prey vectors were transformed into yeast cells harboring the pAbAi-*HfFT1* and pAbAi-*HfFT2* bait constructs. After transformation, colonies were grown on SD/-Leu solid medium containing the optimal concentration of AbA for inhibition and incubated at 30 °C for 3 days. Single colonies were selected and cultured in SD/-Leu liquid medium for 1 day. Serial dilutions of the yeast culture (10, 10^−1^, 10^−2^, 10^−3^) were prepared, and 10 µL of each dilution was spotted onto SD/-Leu solid medium containing the optimal AbA concentration. Interaction was confirmed by culturing in a constant temperature shaking incubator at 30 °C for 3 days.

### 5.4. Analysis of Gene Expression Rhythm

Based on the existing transcriptome data of daylily [27], PCR primers for the *HfGI*, *HfLHY*, and *HfCOL2* genes were designed using Primer Premier 5.0 software (Table 1). The amplified products were subjected to 2% agarose gel electrophoresis, followed by recovery and purification. These products were then ligated into the pGM-T vector to construct recombinant plasmids, which were subsequently transformed into TOP10 competent *Escherichia coli* cells. Transformants were selected and cultured overnight in LB liquid medium containing ampicillin at 37 °C and 200 rpm, and confirmed by PCR. The correctly identified plasmids were sequenced. Plasmids containing the genes were extracted using a plasmid mini-prep kit (TIANGEN, Beijing, China). After measuring their concentration with a spectrophotometer, the plasmids were serially diluted to concentrations of 10^−4^, 10^−5^, 10^−6^, 10^−7^, 10^−8^, and 10^−9^ copies. Using these dilutions as templates, fluorescence quantitative PCR was performed in triplicate, and a standard curve was established.

For detailed steps and calculations, please refer to Livak and Schmittgen (2001). The RT-qPCR reaction was performed using the 2×SuperFast Universal SYBR Master Mix (Beijing Kangweishiji, Beijing, China) in accordance with the manufacturer’s instructions. Each sample was analyzed in triplicate as technical replicates. Primer sequences can be found in Table 2, while RT-qPCR conditions are cited according to the provided guidelines. The reference gene employed in this study was *HfTCTP* from *Hemerocallis* [27]. Gene expression levels were quantified by determining the ratio of its copy number concentration to that of the reference gene *HfTCTP*.

### 5.5. Isolation of HfCOL2, Vector Construction, and Plant Transformation

Based on the existing transcriptome data of daylily [27], the *HfCOL2* gene was initially cloned using the reverse transcriptase polymerase chain reaction (RT-PCR) with the primers *HfCOL2*-F1 and *HfCOL2*-R1 (Table 1). Three independent fragments were sequenced by Qingke (Beijing, China), and the resulting consensus sequence served as the template for amplifying the open reading frame (ORF) of *HfCOL2* using the primer pair 35S:*HfCOL2*-F/35S:*HfCOL2*-R (Table 1). The amplified fragment was inserted into the pBI1304 binary vector via an integrated cloning technique (Thermo Fisher, Shanghai, China), generating the recombinant plasmid pBI1304-*HfCOL2*. This plasmid was then transformed into EHA105 strains and subsequently introduced into tobacco via Agrobacterium-mediated leaf disk transformation. The infected leaf explants were co-cultivated on MS medium supplemented with 0.1 mg/L NAA and 0.05 mg/L 6-BA for 3 days. After co-cultivation, the leaf disks were transferred to selection MS medium containing 0.5 mg/L 6-BA, 0.1 mg/L NAA, 500 mg/L cefotaxime, and 5 mg/L Hygromycin B. Shoots measuring 2 to 3 cm were then transferred to rooting 1/2 MS medium supplemented with 0.2 mg/L NAA and 100 mg/L timentin. Plantlets with well-developed roots were transplanted to soil and cultivated at 25 °C under a 16/8 h (light/dark) photoperiod. To verify and obtain the overexpression lines, we extracted genomic DNA from fully developed leaves of the transgenic plants. We then performed PCR using 35S:*HfCOL2*-F/35S:*HfCOL2*-R primers and the extracted DNA as templates for amplification and detection, and at least 10 independent overexpression lines were obtained.

The seeds of wild-type plants and T1 transgenic plants overexpressing *HfCOL2* were sown in pots with a diameter of 15 cm. All plants were grown under identical conditions, including the same medium composition (2.5 kg per pot, with a ratio of nutrient soil to vermiculite of 3:1) and consistent watering amounts. Phenotypic changes and flowering time at each developmental stage were systematically observed and recorded. Three transgenic lines and wild-type plants were selected for detailed observation. Flower buds from these plants during the flowering period were monitored from initial opening until complete senescence. Daily photographs were taken to document flower development. For each plant, three flowers were observed on three separate dates, capturing their progression from the initial opening stage to the senescence stage.

## Figures and Tables

**Table 1:** Specific primers for gene isolation.

Gene Name	Primer Name	Primer Sequence (5′ → 3′)	Accession Number
*HfCOL*2	*HfCOL*2-F1	GAGGCACTTTTCGGTGAGGA	PQ149922
	*HfCOL*2-R1	GCACCCTCGCTTCTCTATCC	
*HfCOL4*	*HfCOL14*-F1	TCCAAACTCAAACTGAACCATATCC	PQ149925
	*HfCOL14*-R1	TTACTACAAATTATCCGAACACCGG	
*HfCOL9*	*HfCOL9*-F1	CTTCTTCAATGTGGTCGCAGAGTTA	PQ149923
	*HfCOL9*-R1	ACCTGACTGGGCAAAAGTTAGACCT	
*HfCOL14*	*HfCOL4*-F1	TCAAACAGCGTCAAAAGCGACTGAC	PQ149924
	*HfCOL4*-R1	TGATGGTTGGTACATGGGCCACTGG	
*HfCOL16*	*HfCOL16*-F1	GCTCCCAGATTGCCCTTTAT	PQ149926
	*HfCOL16*-R1	ACCGGGCTTGCTAGAGT	
*HfGI*	*HfGI*-F1	ATGTCTACTTGTGCTTCTTGTCATGAG	PQ149920
	*HfGI*-R1	CTATCATCATCAGATCTGAGACACCG	
*HfLHY*	*HfLHY*-F1	ATGGATACGAAAACGTTGGGGGATGA	PQ149921
	*HfLHY*-R1	CTGCAAAGACGGTTGTTTGTGAAAATG	
*HfCOL*2	35S*:HfCOL*2-F	agaacacgggggactcttgaccatgg ATGATGAGGCATTGCGATTCGTG	
	35S*:HfCOL*2-R	tgaaaagttcttctcctttactagt TTAGAATGAGGGCACAATCC	

**Table 2:** Specific primers for gene expression analysis.

Gene Name	Primer Name	Primer Sequence (5′ → 3′)	Fragment Length
*HfCOL*2	*HfCOL*2-F	GAGGCACTTTTCGGTGAGGA	240
	*HfCOL*2-R	GCACCCTCGCTTCTCTATCC	
*HfGI*	*HfGI*-F	CTTGCGGCCTCTATCTTCGT	233
	*HfGI*-R	CTACAACTTGTCGGGGGCTT	
*HfLHY*	*HfLHY*-F	TGTGAGTTTTGTGGGGAGCA	252
	*HfLHY*-R	AAAGAGTTCGAGAGTGGCGG	
*HfTCTP*	*HfTCTP*-F	GGTTGCTCCTGAAGCCTGAA	211
	*HfTCTP*-R	TCAGCGGAAGGAGGAGAAGA	

## Data Availability

The datasets analyzed during the current study are available from the corresponding author on reasonable request. The data are not publicly available due to specific contractual obligations with funding entities and in alignment with scheduled follow-up studies.
